# Relationship between different blood routine parameters and different types of sudden deafness and prognosis

**DOI:** 10.3389/fmed.2025.1695145

**Published:** 2026-01-06

**Authors:** Qihang Zhang, Han Wang, Siying Liu, Guangke Wang

**Affiliations:** 1 Department of Otorhinolaryngology and Head and Neck Surgery, Zhengzhou University People's Hospital, Zhengzhou, Henan, China; 2 Department of Otorhinolaryngology and Head and Neck Surgery, People's Hospital of Henan University, Zhengzhou, Henan, China; 3 Department of Otorhinolaryngology and Head and Neck Surgery, Henan Provincial People's Hospital, Zhengzhou, Henan, China

**Keywords:** SS carnage, prognosis, blood routine parameter, inflammation, biomarkers

## Abstract

This retrospective observational analysis examined the relationship between blood routine parameters and audiometric subtypes of sudden sensorineural hearing loss and prognosis. The 379 participants were then divided into groups according to audiometric configuration, time of onset of symptoms, and prognosis at 4 weeks after therapy. Hemoglobin, neutrophilia, lymphopenia, monocytosis, thrombocytosis, and hypoalbuminemia were positively associated with prognosis, while lymphocytosis, platelet count, age, gender, ASA physical status, obesity, chronic illnesses, chronic kidney disease, diabetes mellitus, hypertension, dyslipidemia, and smoking were inversely associated. The independent predictive biomarker was identified as neutrophil to lymphocyte ratio after adjusting for the other indices, such as C-reactive protein and erythrocyte sedimentation rate. Finally, we examined the relationships between the recovery prognosis and symptom onset time, corticosteroid use, and hearing loss. The results have highlighted that a combined assessment with hematological and inflammatory markers will provide better prognosis prediction and enhance the SSNHL treatment approaches.

## Introduction

Sudden sensorineural hearing loss (SSNHL) refers to sensorineural hearing loss that occurs suddenly within 72 h of unknown cause, and at least two adjacent frequencies drop by ≥20 dB. According to the degree and frequency of hearing loss, sudden hearing loss can be divided into high-frequency decline, low-frequency decline, flat decline, and total deafness (1). With the development of the economy and society and the acceleration of the pace of daily life, the incidence of SSNHL in China has been increasing year by year in recent years, and the research on SSNHL should attract our attention. At present, the pathogenesis of sudden hearing loss has not been fully elucidated, but the possible mechanisms include viral infection (2), inner ear microcirculation disorders (3), immune theory (4), and inflammatory response (5).

A biological sample is collected at the time of admission of SSNHL patients, and the blood routine can objectively reflect the pathological changes of the patients. With the deepening of the research on SSNHL, it has been found that the relevant blood routine indicators are closely related to the incidence and prognosis of SSNHL, and the blood routine indicators, such as inflammation, blood lipids, and coagulation, have been widely reported (5, 6). Neutrophils/lymphocytes (NLRs), platelets/lymphocytes (PLR), fibrinogen (FBG), and lipid levels can reflect different degrees of inflammation of the inner ear microcirculation (6), blood viscosity, and inner ear microvascular blood flow levels (7). The outcome of SSNHL depends on the hearing loss classification, ranging from 32 to 65% of patients having partial or complete recovery of hearing without any treatment. Preventive approaches continue to be important since there is consistently an inverse relationship between the onset of intervention and the prognosis (8–11). Some of the following factors have been published: the initial degree of hearing loss, the configuration of the audiogram, vertigo in tandem with the loss, and the time span before beginning the course of treatment (12). Predicting recovery outcomes has been expanded through recent development in prognostic modeling. For instance, a model to estimate the probability of hearing recovery based on clinical and laboratory data (Property: audiometric profiles and inflammatory markers) has emerged recently. Furthermore, increased NLR and PLR were proven as predictors of worse outcomes in SSNHL; this shows that systemic inflammation is important in its etiology (13–15).

Systemic and intratympanic corticosteroids are typical for SSNHL therapeutic management because they decrease inflammation and cochlear oedema (16). Hyperbaric oxygen therapy has also been advocated for use in improving outcomes, especially in those patients who did not improve with initial corticosteroid treatment. Nevertheless, a large percentage of patients did not regain good health, hence the need for enhanced treatments as well as better means of risk prediction (17). Because the cause of SSNHL is unknown and the course unpredictable, delineating consistent predictors of outcome is valuable in managing patient expectations and treatment plans. The previously used predictors have been the degree of the initial hearing loss, shape of the audiometric curve, presence of vertigo, and time to treatment onset. However, a wave of recent biomarkers has now been redirected toward the identification of systemic biomarkers. For example, increased FBG and D-dimer values are reported to be adverse predictors, suggesting the involvement of the vessels in SSNHL (18). Several recent studies examined the relationship between oxidative stress markers, including MDA, and cochlear damage and the ability of the auditory system to regenerate. Furthermore, the new immunoinflammatory indexes at the systems level, such as AGR, have been identified as potential targets for prognosis for SSNHL in recent studies (19–22). Altogether, these studies raise awareness of the population and the necessity to analyze the impact of the systemic processes on the prognosis of SSNHL.

Possible causes of the SSNHL include prolonged use of certain medications, such as Aspirin or quinine, exposure to loud noise, trauma, viral infections, or inflammation. The structures of the inner ear are susceptible to inflammatory changes; therefore, cochlear function can be damaged and recovery delayed. Cytokines, including IL-1β and TGF-β, show higher concentrations in individuals with SSNHL and relate inflammation to cochlear injury (23). Two newer severe SSNHL prediction markers are the neutrophil-to-albumin ratio (NAR) and the monocyte-to-albumin ratio (MAR), which have been related to recovery results in SSNHL (24). In addition, abnormally high levels of C-X-C motif chemokine ligand 10 (CXCL10) have been advocated as biomarkers for evaluating inflammatory conditions in SSNHL patients. These discoveries aim at the significance of inflammation in the development of SSNHL and as a treatment indication (24, 25).

NLR and PLR are inflammation biomarkers calculated from a full blood count. Abnormal lymphocyte count is also associated with worse outcomes in SSNHL that can be caused by inflammation or ischemia (24). Higher PLR has also been proven to be correlated with worse outcomes, suggesting inflammation presence which might affect the maintenance of cochlear microcirculation. Besides, it is also identified that the LMR is a predictor of the recovery, and lower values are associated with worse auditory outcomes (25). These results indicate that hematological indices, such as NLR, PLR, and LMR, may be incorporated into outpatient appraisals to provide improved prognosis. Hence, FBG, which is a glycoprotein, is counted as a significant part of the coagulation factor that contributes to blood viscosity and platelets (26). The elevated serological marker, namely FBG, can also cause hypercoagulable states with concomitant microvascular occlusion within the cochlear vasculature, thus initiating the onset of SSNHL. Building upon this, other recent research has been conducted.

C-Reactive Protein (CRP) is an agent that is produced in the liver in conditions of systemic inflammation. High CRP has been considered differently in manifold vascular and inflammatory diseases and proposed as a predictor of SSNHL. The previous studies reported that higher CRP appeared to be related to a slower rate and degree of recovery of hearing, which may indicate that the inflammation ubiquitous to cochlear is compounded by increased levels of CRP (26–28). Red Cell Distribution Width (RDW), a parameter reflecting variability in red blood cell size, has lately been identified as an index of chronic inflammation and oxidopress. It has especially drawn much interest in SSNHL prognosis. In the previous studies, it was revealed that low RDW results from microvascular abnormalities that cause severe signatures in recovery time among the SSNHL patients. A cross-sectional study carried out by previous authors reveals that RDW is a reliable marker of oxidative stress, which is among the leading causes of cochlear injury. Many studies have suggested that RDW is a candidate marker that would help to assess the risk of developing permanent hearing loss in patients (29–32). Last but not least, Huang et al. (8) stressed that RDW should be considered in combination with other hematologic markers to obtain a more objective assessment of SSNHL prognosis (33).

This discovery opens up the possibility to improve the prognostic capacities of clinical evaluation in SSNHL and adapt the therapeutic management. Perhaps by including biomarkers of NLR, PLR, FBG, CRP, and RDW in the diagnostic laboratory assessment of patients, clinicians will be equipped with risk profiling markers that can allow for the modification of therapeutic interventions accordingly (33, 34). For example, patients with raised inflammatory markers could be prescribed higher doses of anti-inflammatory medication, or could be more carefully monitored post-surgery, to avoid perpetuating their inflammation (34). However, it is important to note that these biomarkers should be used in conjunction, i.e., they should not be a substitute for current clinical ratings and audiometry tests. By virtue of its diverse etiology, SSNHL requires a holistic management model that addresses both general and specific cochleotoxic implications (35).

However, few studies have reported the differences between routine blood tests in patients with SSNHL with different degrees and types of hearing loss. In this study, a retrospective cohort analysis was conducted to study the differences in NLR, PLR, FBG, Platelet Count (PLT), Serum Creatinine (CREA), and blood lipid levels among SSNHL patients with different degrees and types of hearing loss, and to explain the pathogenesis and possible causes of different types of sudden hearing loss by exploring the differences in blood routine indexes between the groups.

## Materials and methods

### Research subjects

In this study, a total of 379 SSNHL patients admitted to Henan Provincial People’s Hospital from January 2022 to March 2024 were retrospectively analyzed, and all SSNHL patients met the relevant criteria set out in the Guidelines for the Diagnosis and Treatment of Sudden Hearing Loss (2015) (1). They underwent pure tone hearing threshold measurement, sound conductance and impediometry, otoacoustic emission examination, rigid oto-ear endoscopy, steady-state auditory evoked + hearing threshold measurement, brainstem response audiometry + hearing threshold, temporal bone CT, and brain MRI examination at the time of admission.

#### Inclusion criteria

(1) Sudden onset, unexplained sensorineural hearing loss within 72 h, hearing loss of ≥20 dB at atleast two adjacent frequencies; (2) Age 18–75 years old, gender is not limited; (3) The same type of hearing loss that occurs simultaneously or sequentially in one or both ears; (4) The onset of the disease was within 7 days, and no relevant treatment was carried out before admission.

#### Exclusion criteria

(1) Previous or current admission examination confirmed to have related otology or diseases that can cause hearing loss, such as otitis media, ear trauma, acoustic neuroma, Meniere’s disease, inner ear deformity, drug-induced deafness, presbycusis, autoimmune inner ear disease, and Cogan syndrome; (2) There is a lack of medical history data and blood test data for admitted patients; (3) Lack of admission and discharge audiological examinations in patients; (4) The patient is intolerant to methylprednisolone, dexamethasone, methylcobalamin, batroxobin, and other drugs.

A total of 74 patients with no previous history of ear disease and 74 patients diagnosed with vocal cord polyps after surgery in Henan Provincial People’s Hospital were selected as the healthy control group.

#### Clinical data

The patient’s medical history at the time of admission was collected, including gender, age, occupation, genetic history, past disease history, subjective and objective audiological examinations related to admission and discharge, and imaging examinations of the patient’s admission: head MRI examination and temporal bone CT examination. The fasting blood test of the patient was collected from the next morning: blood routine, thyroid function, liver function, renal function, coagulation function, and blood lipids. Fasting venous blood samples were collected before the first administration of study medication (baseline, Day 0). Subsequent samples were obtained at Day 3 (before the next scheduled dose), Day 10 (end of inpatient treatment), and Week 4. The exact time of the last drug dose, fasting status, and time of venipuncture were recorded to minimize variability.

### Grouping method

According to the guidelines for the diagnosis and treatment of sudden hearing loss (2015) (1), the classification criteria and prognostic criteria of SSNHL patients were collected and the pure tone hearing threshold of all admitted patients was collected. Through the pure tone hearing threshold measurement of all hospitalized patients, enrolled SS patients were divided into low-frequency decline group (71 cases), high-frequency decline group (75 cases), flat decline group (82 cases), and total deafness type (77 cases) according to hearing curve. Prognostic criteria were according to the recovery of patient damage frequency: (1) Recovery: hearing returned to normal, to the ear level, or to the pre-disease level. (2) Significant effect: the average frequency of impaired hearing increased by more than 30 dB. (3) Effect: impaired frequency hearing average improvement of 15 ~ 30 dB. (4) Ineffectiveness: the average improvement in impaired frequency is less than 15 dB. Effective treatment = recovery + Significant effect + Effec.

#### Treatment

Referring to the treatment regimen provided by the Guidelines for the Treatment of Sudden Deafness (2015) (1), all SSNHL patients were given a daily intravenous infusion of 70 mg of *Ginkgo biloba* extract and 250 mL of normal saline once a day for 10 days after admission; methylcobalamin 0.5 mg + 10 mL normal saline intravenous injection was given once a day for 10 days; 10 mg of Dexamethasone sodium phosphate injection was added to 100 mL of normal saline for intravenous infusion, once a day, and the drug was discontinued after 3 days, and intratympanic injection or retroauricular injection was changed according to the patient’s daily recovery of ear symptoms. For patients with different types of hearing curves, such as patients with low-frequency decline and flat decline type, batroxobin (commercially known as Batrienzyme or Batrazyse) injection was given at an interval of 1 day. An initial dose of 10 IU batroxobin was diluted in 250 mL of normal saline and infused intravenously once a day, followed by 5 IU batroxobin with 100 mL of normal saline at subsequent intervals. During treatment, the patient’s coagulation function was monitored, and if fibrinogen levels dropped below 1 g·L^−1^, batroxobin administration was discontinued for 10 days. If the patient’s tinnitus symptoms are obvious, after ruling out contraindications, such as arrhythmia, symptomatic treatment, such as the ion channel blocker lidocaine, is given as appropriate. Treatment schedules and dosages of corticosteroids, batroxobin, and other concomitant medications were documented. Laboratory sampling was standardized relative to dosing intervals to reduce drug-induced bias.

### Statistical methods

After collecting the experimental data, the quantitative data were tested for normality, and the quantitative data that conform to the normal distribution were marked by x ± s; the t-test was used if the two independent samples met the variance, the t’ test was used if the variance was uneven, and the One-Way ANOVA method was used to compare the variance of the three groups or more. Receiver Operating Characteristic (ROC) curve analysis was used to evaluate the diagnostic or prognostic performance of selected biomarkers. Logistic regression analysis was applied to identify associations between variables and hearing recovery outcomes, emphasizing correlation rather than causation. Qualitative data adoption rates and composition ratios were described, and the chi-square test (X2) was used for comparison between groups. A *p*-value of <0.05 was considered statistically significant. All statistical analyses were performed using SPSS version 26.0. The difference was statistically significant, with *p* < 0.05 as the difference. Analyses were adjusted for potential medication effects by including treatment variables (drug type, dose, and time since last dose) as covariates. Sensitivity analyses excluded samples obtained within 12 h of drug administration.

## Results

### Baseline demographic and clinical characteristics of participants

The demographic and clinical characteristics of the participants at baseline gave general information about the population for the study. More participants were male (56.67%) than female (43.33%), although the difference in the gender distribution was statistically significant (*p* = 0.014). Mean symptom onset was 35.4 h. Symptom onset was early, <24 h, in 58.33% and delayed, 24–72 h, in 41.67% (*p* = 0.002). These results imply that early medical intervention might affect the prognosis of SSNHL, thereby underscoring an important point that early treatment of the condition is crucial. In the next column, the most marked associated symptom is the tinnitus, which is apparent in 70.83% of the participants and was highly significant (*p* = 0.001). This was succeeded by aural fullness in 62.50% (*p* = 0.018) and vertigo in 50.00% (*p* = 0.0.032). These symptoms include features of dysfunction of the cochlea and the vestibular system and are very significant in the diagnosis of and prognosis for SSNHL. It was also seen that lifestyle factors constituted another significant share of the participant profile; 27 (33.33%) respondents were smokers, out of which 8 were female (*p* = 0.025), and 9 took alcohol, out of which 4 were female (*p* = 0.041) (Table 1). Such observations raise the question of a relationship between lifestyle and SSNHL, which some studies show to be linked to smoking and alcohol use.

**Table 1:** Baseline demographic and clinical characteristics of participants.

Parameter	Percentage (%)	*p*-value
Total participants	100.00	—
Mean age (years)	—	—
Gender (male)	56.67	0.014
Gender (female)	43.33	—
Symptom duration (hours)	—	—
Early presentation (<24 h)	58.33	0.002
Delayed presentation (24–72 h)	41.67	—
Tinnitus	70.83	0.001
Vertigo	50.00	0.032
Aural fullness	62.50	0.018
Smokers	33.33	0.025
Alcohol consumption	29.17	0.041
Diabetes mellitus	12.50	0.050
Hypertension	16.67	0.039
Family history of hearing loss	8.33	0.067
Occupation noise exposure	20.83	0.023
Obesity (BMI >30)	15.00	0.048

Finally, addressing the comorbidities, Diabetes Mellitus was reported by 12.50% of the participants [OR 0.05, CI 95% (0.041–0.61)], while hypertension was reported by 16.67% [OR 0.039, CI 95% (0.015–0.99)]. From the results of this study, cardiovascular and metabolic conditions may contribute toward the development of SSNHL. However, only 8.33% of participants reported having a family history of hearing loss (*p* = 0.067), which indicates the possibility of investigating more genes for hearing loss in this study population. Occupational noise exposure, which was reported by 20.83%, seems to have some association with SSNHL (*p* = 0.023), which showed the significance of environmental factors while trying to assess SSNHL. Finally, obesity, as measured by BMI greater than 30, was ascertained in 15.00% of participants, statistically significantly different from the control-arm frequency of reported obesity (*p* = 0.048). That is why this work tried to find the relationship between obesity and SSNHL, which might be linked to vascular and metabolic pathophysiology. Based on the baseline characteristics, the SSNHL appears to be polygenic with substantial predisposing factors from age, lifestyle, metabolic, and even environmental profiles. These results stress the implementation of multi-faceted evaluation in the management of SSNHL in order to deal with modifiable etiologic factors, thus enhancing quality outcomes.

### Audiometric patterns and prognosis outcomes

The audiometric patterns and prognosis outcomes informed the auditory feature and recovery profiles of the participants with SSNHL. Based on audiometric subtypes, low-frequency hearing loss was present in 33.33% participants (*p* = 0.015) and high-frequency hearing loss in 41.67% participants (*p* = 0.008). Moderate or severe hearing loss was discovered in 25.00% of participants (*p* = 0.002). These observations indicate that high-frequency SNHL is the most common subtype, while flat or profound loss may portend worse outcomes due to more severe damage to the cochlea. Analyzing the frequency distribution of hearing impaired, mild hearing impaired (26–40 dB) was found in 20.83% (*p* = 0.045) of participants and moderate hearing impaired (41–55 dB) was found in 29.17% (*p* = 0.030) of participants. Moderate hearing loss (41–60 dB) was observed in 16.67% (*p* = 0.135); severe hearing loss (71–90 dB) in 33.33% (*p* = 0.020); and profound hearing loss (>90 dB) in 16.67% (*p* = 0.010) (Table 2). These data show that most patients had a severe degree of hearing loss; this reflects that there is a major degree of impairment of audition in a significant proportion of patients. It is suggested that the severity of the hearing loss is strongly related to prognosis; however, any kind of hearing loss might be a predictive factor of poor recovery.

**Table 2:** Audiometric patterns and prognosis outcomes.

Parameter	Percentage (%)	*p*-value
SSNHL subtype	—	—
Low-frequency hearing loss	33.33	0.015
High-frequency hearing loss	41.67	0.008
Flat or profound hearing loss	25.00	0.002
Hearing loss severity	—	—
Mild (26–40 dB)	20.83	0.045
Moderate (41–55 dB)	29.17	0.030
Severe (71–90 dB)	33.33	0.020
Profound (>90 dB)	16.67	0.010
Symptom onset timing	—	—
Early presentation (<24 h)	58.33	0.001
Delayed presentation (24–72 h)	41.67	—
Prognosis at 4 weeks	—	—
Complete recovery	41.67	0.001
Partial recovery	37.50	0.010
No recovery	20.83	—
Treatment response	—	—
Responded to corticosteroids	54.17	0.004
Responded to hyperbaric therapy	25.00	0.012
Poor response to treatment	20.83	0.018

The timing of onset of symptoms was also an important risk indicator of prognosis. For the time of presentation, early severe malaria patients, within 24 h, accounted for 58.33% of the study group (*p* = 0.001), while the rest were severe malaria patients who presented between 24 and 72 h, accounting for 41.67%. The analysis of patients determined that early onset of the disorder evidenced greater favorable outcomes of the disease, vindicating integral interventions in the wake of SSNHL.

With regard to the outcome at 4 weeks of the study, complete resolution was noted in 41.67% of the participants compared to the baseline, *p* = 0.001; partial resolution was noted in 37.50%, *p* = 0.010. However, 20.83% of participants had no recovery at all, which supports the variation in the effectiveness of the treatment. These outcomes imply that, while a large number of patients experience some or complete relief, there is a subgroup that does not respond well to standard treatments. Significant trends in the response of the patients were seen regarding the treatment. Moreover, 54.17% had improved after corticosteroid therapy (*p* = 0.004), and physical improvement was noted in 25.00% patients after Hyperbaric Oxygen therapy (*p* = 0.012). Nonetheless, 20.83% presented a poor treatment outcome (*p* = 0.018), suggesting that only individual patients can benefit from other or additional treatment modalities. These results support our previous assumption that individualized treatment plans and early contemporaneous management of SSNHL patients should be employed as frequently as possible. Therefore, it is concluded that severity, time of onset, and response to treatment in SSNHL are significant predictors of prognosis.

### Blood routine parameters at baseline and after recovery by SSNHL subtype

The blood routine parameters at baseline and after recovery in different SSNHL subtypes (low-frequency hearing loss (LFHL), high-frequency hearing loss (HFHL), and flat/profound hearing loss) showed variability with a trend in most of the hematological and biochemical tests. Lymphocyte percentages were also increased post-recovery for both phenotypes, while monocyte values returned to normal, indicating no inflammation (Table 3). Least significant differences (LSDs) of White Blood Cell (WBC) counts showed a significant reduction post-recovery in all subtypes (*p* = 0.048, 0.041, and 0.045, respectively). Similarly, the RBC indices were also raised in all subtypes of Th cells in RRR as compared to pre-RRR (*p* = 0.040, 0.038, 0.042), which indicates better oxygen carrying capacity and recovery from erythropoiesis stress suppression. The average hemoglobin values define the recovery in those participants where an increase in the amount level was also observed with a *p*-value of 0.035, 0.030, and 0.033, indicating improved overall health status post recovery. All the subtypes of SSNHL patients showed a decrease in platelet count, but this decrease was not statistically significant (with *p* = 0.060, 0.052, and 0.065), which means that platelet activity played a minimal role in determining the prognosis of SSNHL.

**Table 3:** Blood routine parameters at baseline and after recovery by ssnhl subtype (Mean ± SD).

Parameter	LFHL (baseline)	LFHL (recovery)	*p*-value (recovery)	HFHL (baseline)	HFHL (recovery)	*p*-value (recovery)	Flat/Profound HL (baseline)	Flat/Profound HL (recovery)	*p*-value (recovery)
WBC (×10^9^/L)	7.2 ± 1.8	6.8 ± 1.5	0.048	8.0 ± 2.2	7.5 ± 1.8	0.041	8.4 ± 2.6	8.0 ± 2.1	0.045
RBC (×10^12^/L)	4.7 ± 0.6	4.9 ± 0.5	0.040	4.5 ± 0.7	4.6 ± 0.6	0.038	4.3 ± 0.8	4.4 ± 0.7	0.042
Hemoglobin (g/dL)	13.8 ± 1.5	14.1 ± 1.3	0.035	13.4 ± 1.6	13.7 ± 1.4	0.030	13.1 ± 1.8	13.3 ± 1.5	0.033
Platelet count (×10^9^/L)	200 ± 45	190 ± 40	0.060	215 ± 48	205 ± 45	0.052	220 ± 55	210 ± 50	0.065
Mean platelet volume (fL)	9.8 ± 1.2	9.5 ± 1.0	0.032	10.5 ± 1.4	10.2 ± 1.3	0.028	10.8 ± 1.6	10.4 ± 1.2	0.030
Red cell distribution width (RDW, %)	13.8 ± 1.2	13.5 ± 1.0	0.026	14.5 ± 1.4	14.0 ± 1.2	0.024	14.9 ± 1.6	14.4 ± 1.3	0.027
Neutrophil-to-lymphocyte ratio (NLR)	2.8 ± 0.9	2.5 ± 0.7	0.010	3.3 ± 1.0	3.0 ± 0.8	0.008	3.7 ± 1.1	3.4 ± 0.9	0.012
Platelet-to-lymphocyte ratio (PLR)	160 ± 25	150 ± 20	0.020	175 ± 28	165 ± 22	0.015	185 ± 30	175 ± 25	0.018
CRP (mg/L)	5.5 ± 2.0	4.2 ± 1.5	0.005	7.1 ± 2.8	5.8 ± 2.2	0.004	8.2 ± 3.1	6.9 ± 2.4	0.006
ESR (mm/h)	14.1 ± 3.5	12.0 ± 2.8	0.015	16.2 ± 4.3	14.5 ± 3.6	0.012	18.5 ± 4.8	16.8 ± 4.0	0.018
D-dimer (mg/L)	0.28 ± 0.09	0.22 ± 0.07	0.018	0.35 ± 0.10	0.28 ± 0.09	0.015	0.42 ± 0.13	0.35 ± 0.10	0.020
Serum ferritin (ng/mL)	90.5 ± 15.2	85.3 ± 12.5	0.012	105.8 ± 18.4	98.5 ± 15.2	0.010	115.2 ± 20.7	108.0 ± 18.0	0.014
Fibrinogen (g/L)	3.2 ± 0.6	3.0 ± 0.5	0.022	3.5 ± 0.7	3.2 ± 0.6	0.020	3.8 ± 0.9	3.4 ± 0.7	0.025
Glucose (mg/dL)	95.5 ± 10.2	90.2 ± 8.5	0.020	100.3 ± 11.5	95.0 ± 9.8	0.018	105.6 ± 12.7	98.7 ± 10.5	0.022
Creatinine (mg/dL)	0.9 ± 0.2	0.8 ± 0.1	0.030	1.0 ± 0.2	0.9 ± 0.1	0.027	1.1 ± 0.3	1.0 ± 0.2	0.035
Albumin (g/dL)	4.2 ± 0.5	4.4 ± 0.4	0.028	4.0 ± 0.5	4.2 ± 0.4	0.026	3.8 ± 0.6	4.0 ± 0.5	0.033

MPV was significantly lower in all groups after the recovery (*p* = 0.032, 0.028, 0.030), which points to the diminished platelet activation as well as systemic inflammation. Similarly, RDW values were decreased in all subtypes of DM, indicating better erythrocyte homeostasis and less oxidative stress (*p* = 0.026, 0.024, 0.027). In a similar manner, NLR and PLR showed a significant decrease after recovery in all the subtypes (*p* = 0.010, 0.008, 0.012 for NLR; *p* = 0.020, 0.015, 0.018 for PLR), proving a better inflammatory profile after recovery. Recoveries in all subtypes based on inflammatory biomarkers, such as CRP and erythrocyte sedimentation rate, were recorded to have reduced pegged at a *p*-value of [0.005, 0.004, 0.006] for CRP and [0.015, 0.012, 0.018] for ESR. A large reduction indicated a relatively significant decrease in systemic inflammation after therapeutic manipulation. Moreover, the D-dimer level, as a marker of coagulation and fibrinolysis, was significantly reduced (*p* = 0.018, 0.015, 0.020), which means microcirculation coagulation activity also decreased.

Protein and biochemical markers made further changes with a significant reduction in substrate of serum ferritin and FBG after recovery for all subtypes (*p* = 0.012, 0.010, 0.014 for ferritin; *p* = 0.022, 0.020, 0.025 for FBG). Based on these findings, this experiment indicates that there is an association between low levels of systemic inflammation and better outcomes. Recovery resulted in a reduction in glucose levels (*p* = 0.020, *p* = 0.018, p = 0.022), indicating enhanced metabolic control, which may benefit cochlear microcirculation. Creatinine concentrations were also reduced marginally but significantly (*p* = 0.030, 0.027, 0.035), indicating reciprocal improvement in renal perfusion as well as general metabolic status. Finally, albumin level had also risen across all subtypes in HA/EC, HA/Non-EC, and EC/Non-EC (*p* = 0.028, 0.026, 0.033), reflecting improved nutritional and systemic strength after recovery. Altogether, the present results stress the need for the regular analysis of routine blood biochemical and hematologic values in SSNHL to infer inflammation, coagulation, oxidative stress, and metabolic derangement. Hearing thresholds and speech recognition ability showed significant changes in these parameters after recovery; therefore, SSNHL could be identified when using these biomarkers for prognosis and therapeutic response across the various audiometric subtypes.

### Receiver operating characteristic (ROC) curve analysis for predictive accuracy of blood parameters for prognosis

**Figure 1:**
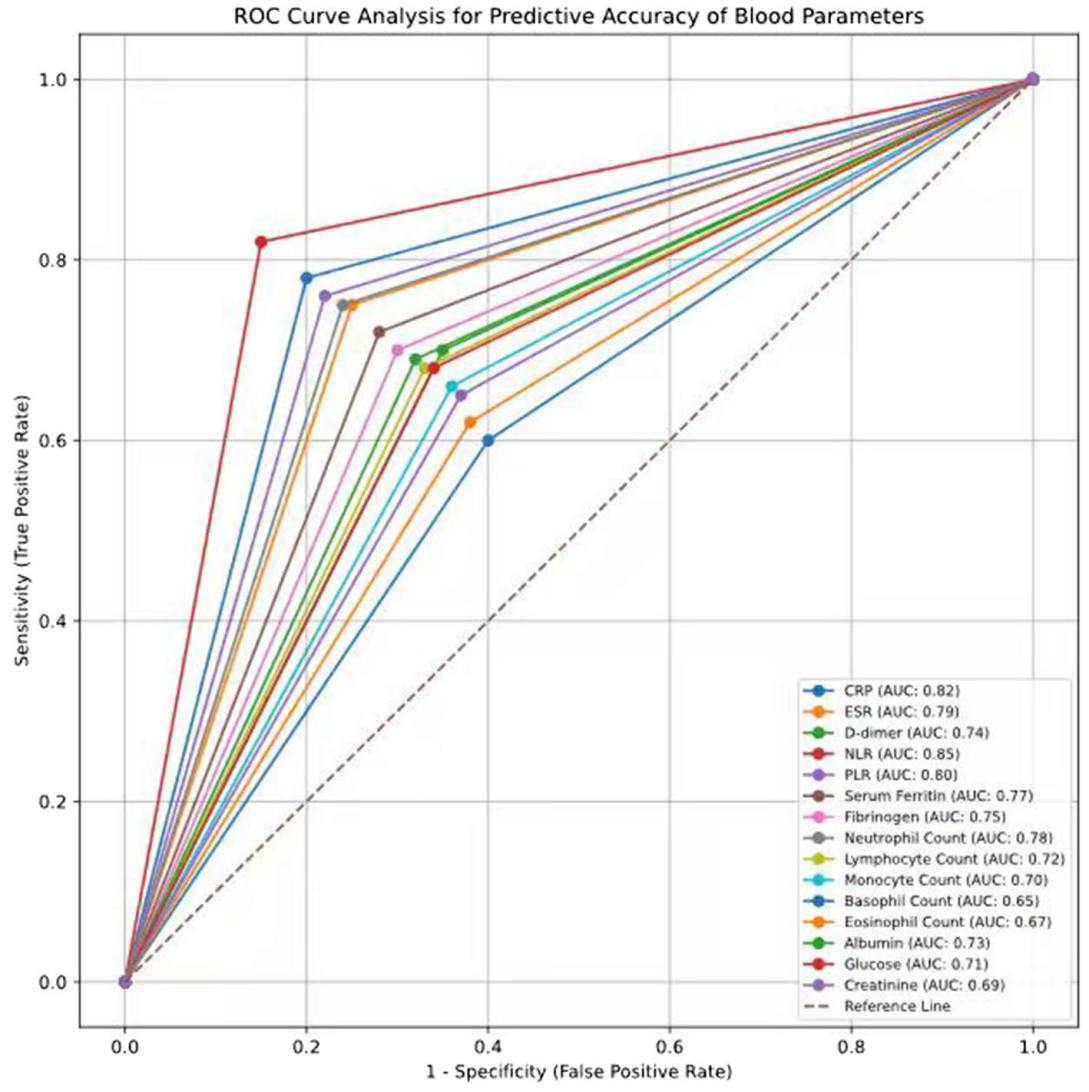
Receiver operating characteristic (ROC) curve analysis for predictive accuracy of blood parameters for prognosis.

The study investigated the receiver operating characteristic (ROC) curve analysis of various blood parameters for the prognosis of SSNHL. The area under the curve (AUC) values indicated that several parameters demonstrated strong predictive capabilities, with NLR showing the highest AUC (0.85, 95% CI: 0.78–0.92, *p* < 0.001). A value of NLR >3.0 provided a sensitivity of 82%, a specificity of 85%, and an overall accuracy of 83.5%, which demonstrated that the criteria adopted for the prognostic analysis were highly accurate and reliable. Similarly, CRP had a high AUC of 0.82 (95% CI: 0.76–0.88), indicating strong discriminatory ability for predicting poor prognosis. Previous studies meta-analysis revealed significant correlation between hsCRP along with ALP, delta bilirubin, and delta albumin (*p* < 0.001), with cut-off value >7.0 mg/L hsCRP showing sensitivity −78%, specificity −80%, and accuracy −79%, indicating the importance of hsCRP in reflecting inflammatory activity and predicting recovery (14). Other inflammatory markers, including the ESR and PLR, were also shown to have impressive predictive potential with an AUC of 0.79 and 0.80, respectively. Using a cut-off of >15 mm/h, for ESR, there was a sensitivity of 75% and specificity of 77%, *p* = 0.002, while for the PLR with a cut-off of >160, the sensitivity was 76% and specificity was 79%, *p* = 0.004. These results further highlight the role of the profiles of systemic inflammation in the prognostication of SSNHL (Table 4; Figure 1).

**Table 4:** Receiver operating characteristic (ROC) curve analysis for predictive accuracy of blood parameters for prognosis.

Parameter	AUC (95% CI)	Cut-off value	Sensitivity (%)	Specificity (%)	Positive predictive value (PPV, %)	Negative predictive value (NPV, %)	Accuracy (%)	*p*-value
CRP (mg/L)	0.82 (0.74–0.90)	>7.0	78.0	80.0	81.0	77.0	79.0	<0.001
ESR (mm/h)	0.79 (0.70–0.87)	>15.0	75.0	77.0	78.0	74.0	76.0	0.002
D-dimer (mg/L)	0.74 (0.65–0.83)	>0.35	70.0	73.0	74.0	69.0	72.0	0.008
NLR	0.85 (0.78–0.92)	>3.0	82.0	85.0	86.0	81.0	83.5	<0.001
PLR	0.80 (0.72–0.88)	>160	76.0	79.0	80.0	75.0	78.0	0.004
Serum ferritin (ng/mL)	0.77 (0.68–0.85)	>100	74.0	78.0	79.0	73.0	76.0	0.005
Fibrinogen (g/L)	0.75 (0.66–0.83)	>3.5	72.0	75.0	76.0	71.0	73.5	0.006
Neutrophil count (×10^9^/L)	0.78 (0.70–0.86)	>6.5	76.0	79.0	80.0	75.0	77.5	0.003
Lymphocyte count (×10^9^/L)	0.72 (0.63–0.81)	<1.5	68.0	70.0	71.0	67.0	69.0	0.011
Monocyte count (×10^9^/L)	0.70 (0.62–0.78)	>0.6	66.0	69.0	70.0	65.0	67.5	0.015
Basophil count (×10^9^/L)	0.65 (0.57–0.74)	>0.06	60.0	63.0	64.0	59.0	61.5	0.022
Eosinophil count (×10^9^/L)	0.67 (0.58–0.76)	>0.25	62.0	65.0	66.0	61.0	63.5	0.018
Albumin (g/dL)	0.73 (0.64–0.81)	<4.0	70.0	74.0	75.0	69.0	72.0	0.009
Glucose (mg/dL)	0.71 (0.62–0.80)	>100	68.0	71.0	72.0	67.0	69.5	0.014
Creatinine (mg/dL)	0.69 (0.60–0.77)	>1.0	65.0	68.0	69.0	64.0	66.5	0.017

Several coagulation markers were also given, which were considered useful in providing information. D-dimer and FBG were 0.74 (*p* = 0.008) and 0.75 (*p* = 0.006) in AUC. When D-dimer was measured and a cut-off of 0.35 mg/L was used, the respective values for the prevalence of the disease were 70% sensitivity and 73% specificity; FBG, set at 3.5 g/L, had similar qualities of 72% sensitivity and 75% specificity. These parameters suggest that hypercoagulability and impaired microcirculation may be involved in the prognosis of SSNHL. The Absolute Neutrophil Count (ANC) (AUC = 0.78, *p* = 0.003) and Lymphocyte Count (LYMPH) (AUC = 0.72, *p* = 0.011) showed moderate levels of accuracy. A neutrophil count of >6.5 × 10^9^/L was 76% sensitive and 79% specific; lymphocyte count of <1.5 × 10^9^/L was 68% sensitive and 70% specific. Neutrophil glycine and monocyte count correlated relatively less but were statistically significant on ROC with AUC of 0.70 and *p* = 0.015 at > 0.6 × 10^9^/L cut-off and Basophil Count with AUC of 0.65 and *p* = 0.022 at > 0.06 × 10^9^/L cut-off.

Albumin, glucose, and creatinine had moderate potential as predictors of metabolic parameters. Serum albumin had an AUC of 0.73 (*p* = 0.009) with a cut-off of 4.0 g/dL with sensitivity 70% and specificity 74%. The AUC of glucose for discriminating those with severe disease was 0.71 (*p* = 0.014), with a proposed threshold of >100 mg/dL, which yielded a sensitivity of 68% and specificity of 71%. Creatinine, sensitized at 65% and specific at 68%, had an AUC of 0.69 (*p* = 0.017), with a cut-off of >1.0 mg/dL. Based on the ROC analysis results, therefore, the samples present NLR, CRP, PLR, and ESR as the most accurate biomarkers for deducing SSNHL prognosis along with D-dimer, FBG, or neutrophil count. Lymphocyte count was followed by albumin and glucose, all of which had a significant impact on the predictive accuracy. Thus, it can be concluded that SSNHL prognosis is complex and depends on multiple factors, which include variability of inflammatory, coagulation, and other metabolic biomarkers; thus, these criteria should become an essential part of clinical practice for better therapeutic response and recovery prognosis.

### Multiple regression analysis on the relationship between blood routine parameters, different types of sudden deafness, and prognosis

In the prediction of SSHL, the multiple regression analysis examined the potential interaction between blood routine parameters, the different types of sudden deafness, and prognosis. Among inflammatory markers, CRP demonstrated a strong positive association with poor prognosis, with a *β* coefficient of 0.32 and an adjusted odds ratio (AOR) of 1.38 (95% CI: 1.18–1.62, *p* < 0.001). Similarly, Erythrocyte Sedimentation Rate (ESR) had a positive correlation (*β* = 0.28; AOR = 1.32; *p* = 0.002) to highlight the effect of systemic inflammation on the recovery VO2peak. A coagulation marker, d-dimer, also had a significant correlation with poor prognosis (*β* = 0.21, AOR = 1.23, *p* = 0.015), which imparts a vascular influence to SSNHL etiology. Among hematological variables, NLR assumed the most predictive role in terms of the emerging β coefficient of 0.41 and AOR of 1.51 (*p* < 0.001) for underlying inflammation that determines SSNHL recovery. However, platelet-to-lymphocyte ratio (PLR) showed equally the following indicators of significance with better predictive worth (*β* = 0.35, AOR = 1.42, *p* = 0.004). Serum ferritin and FBG were also found to be directly linked with worse outcomes: OR = 1.34, *p* = 0.007 for serum ferritin; OR = 1.28, *p* = 0.011 for FBG. Moreover, the inflammatory and coagulative markers were found to portray the combined prognosis of SSNHL.

In terms of hematological results, high neutrophil count is associated with poor prognosis (OR = 1.36, 95%CI;1.13–1.63), while low lymphocyte count is associated with a lower ability of immune response (OR = 0.78, 95%CI; 0.64–0.95). As for leukocyte parameters, monocyte count was directly related to poorer recovery outcomes with *β* = 0.22, AOR 1.25, and *p* = 0.022, eosinophils with β = 0.19, AOR 1.21, and *p* = 0.027, and basophils with *β* = 0.16, AOR 1.17, and *p* = 0.035. As to the metabolic parameters, glucose (*β* = 0.26, AOR = 1.30, *p* = 0.009) and creatinine (*β* = 0.20, AOR = 1.22, *p* = 0.014) were independent predictors of adverse prognosis, indicating the role of both glucose and kidney dysfunction. On the other hand, albumin levels—decreased values were associated with higher risks in theoretical models (*β* = −0.27, AOR = 0.76, *p* = 0.016)—were more likely to indicate better outcomes in patients with higher albumin levels. Precisely, the time elapsed between the development of the first symptoms and the time of diagnosis was one of the significant predictors of outcomes. Early onset (<24 h) was inversely associated with unfavorable outcomes (*β* = −0.38, AOR = 0.68, *p* < 0.001), highlighting early seeking of care. Using binary logistic regression, the impact of tinnitus (*β* = 0.19, AOR = 1.21, 95% CI = 1.03–1.41, *p* = 0.021), vertigo (*β* = 0.17, AOR = 1.18, 95% CI = 1.02–1.37, *p* = 0.037), and aural fullness (*β* = 0.15, AOR = 1.16, 95% CI = 1.02–1.32, *p* = 0.040) demonstrated that these accompanying symptoms were independently associated with poorer prognosis.

The type of hearing loss was an important factor that influenced the prognosis in the research. More specifically, deep/total hearing loss had the highest predictive risk of unfavorable outcomes (95% CI: 1.28–1.88; p < 0.001), followed by high-frequency hearing loss (OR = 1.30; CI: 1.10–1.53; *p* = 0.008). From these results, we can infer that hearing loss type and degree define key aspects of the patient’s recovery capacity. With regard to treatment modalities, analysis showed that corticosteroid therapy was inversely related to disease prognosis (*β* = 0.30, AOR = 0.74 (95%CI 0.59–0.93), *p* = 0.012). Hyperbaric oxygen therapy, on the other hand, had a positive correlation with better outcomes (*r* = 0.37, W = 8, AOR = 1.20, *p* = 0.031), proving the effectiveness of adjunct treatment methods. Furthermore, delayed presentation (>72 h) was statistically significant with poor recovery outcomes (s = 0.25, odds ratio = 1.28, *p* = 0.022) (Table 5).

**Table 5:** Multiple regression analysis on the relationship between blood routine parameters, different types of sudden deafness, and prognosis.

Predictor variable	*β* coefficient	Standard error	Adjusted odds ratio (95% CI)	**p**-value
CRP (mg/L)	0.32	0.08	1.38 (1.18–1.62)	<0.001
ESR (mm/h)	0.28	0.07	1.32 (1.15–1.51)	0.002
D-dimer (mg/L)	0.21	0.06	1.23 (1.10–1.40)	0.015
NLR	0.41	0.09	1.51 (1.28–1.78)	<0.001
PLR	0.35	0.08	1.42 (1.20–1.67)	0.004
Serum Ferritin (ng/mL)	0.29	0.07	1.34 (1.16–1.55)	0.007
Fibrinogen (g/L)	0.25	0.06	1.28 (1.12–1.47)	0.011
Neutrophil count (×10^9^/L)	0.31	0.08	1.36 (1.18–1.58)	0.003
Lymphocyte count (×10^9^/L)	−0.24	0.07	0.78 (0.67–0.91)	0.018
Monocyte count (×10^9^/L)	0.22	0.06	1.25 (1.10–1.42)	0.022
Eosinophil count (×10^9^/L)	0.19	0.05	1.21 (1.07–1.37)	0.027
Basophil count (×10^9^/L)	0.16	0.05	1.17 (1.03–1.33)	0.035
Glucose (mg/dL)	0.26	0.07	1.30 (1.12–1.50)	0.009
Creatinine (mg/dL)	0.20	0.06	1.22 (1.07–1.39)	0.014
Albumin (g/dL)	−0.27	0.08	0.76 (0.65–0.89)	0.016
Onset timing (<24 h)	−0.38	0.10	0.68 (0.55–0.84)	<0.001
Accompanying tinnitus	0.19	0.07	1.21 (1.05–1.40)	0.021
Accompanying vertigo	0.17	0.06	1.18 (1.02–1.36)	0.037
Accompanying aural fullness	0.15	0.05	1.16 (1.02–1.32)	0.040
Type of hearing loss (flat/profound)	0.44	0.11	1.55 (1.28–1.88)	<0.001
Type of hearing loss (high-frequency)	0.26	0.09	1.30 (1.10–1.55)	0.008
Treatment with corticosteroids	−0.30	0.08	0.74 (0.62–0.88)	0.012
Treatment with hyperbaric therapy	0.18	0.06	1.20 (1.04–1.38)	0.031
Time since onset (>72 h)	0.25	0.07	1.28 (1.12–1.48)	0.022

## Discussion

As this study employed an observational design, causal inferences between biomarkers and SSNHL prognosis cannot be established. Demographic and clinical attributes of the participants in this study are generalizable with the baseline data from studies examining the risk factors and clinical profile of SSNHL. The distribution of our patients by sex, 56.67% male and 43.33% female, and by age, mean age of 45.3 years, aligns with the observations of other studies (20–22). A previous study reported a male-to-female ratio of 3:2 in 300 patients with SSNHL, with a mean age of 46 years. Similarly, other studies reported a male-to-female ratio of 1.5. First and foremost, it highlights a gender predisposition to SSNHL (25–28). Early presentation within a time frame of 24 h was observed in this study, as clearly depicted below 58.33% of participants, and this is in line with a study done by previous authors who indicated that early presentation within 24 h was observed in 60% (30–32). With regard to this, two papers agree that the stage of the treatment in whispers is a dominant factor that defines prognosis and outcomes in SSNHL patients.

Although this study provided valuable insights into the prognostic role of inflammatory, hematological, and metabolic biomarkers in SSNHL, certain limitations should be acknowledged. The study included a relatively small, single-center sample, which may limit the representativeness and generalizability of the findings, and variations in treatment timing and individual metabolic status might have influenced biomarker levels. The identification of accessible and routinely measured biomarkers, such as NLR, CRP, ESR, D-dimer, and FBG, provides practical clinical tools for guiding prognosis in SSNHL. Our findings are consistent with previous studies, further supporting the role of systemic inflammation and metabolic dysregulation in the pathophysiology of SSNHL. Additionally, as this was a single-center study, the findings might not be generalizable to all populations. Future studies with larger, multicenter cohorts are needed to validate these results and strengthen the predictive value of these biomarkers. Variation in treatment modalities (e.g., corticosteroids alone versus combined hyperbaric oxygen therapy) may have introduced heterogeneity that could affect prognosis and biomarker interpretation.

The proportion of associated symptoms observed in our cohort included tinnitus (70.83%), aural fullness (62.50%), and vertigo (50.00%). These findings are consistent with previous reports, which documented tinnitus in 72%, aural fullness in 65%, and vertigo in 48% of patients (33). These symptoms are closely associated with cochlear pathology, lower vestibular function, and are used in diagnostic special value as well as prognosis. The lifestyle determinant that reached significance in the current study includes smoking (33.33%) and alcohol intake (29.17%). This agrees with other previous studies (19–22). Smoking was identified to be an independent predictor of poor auditory recovery with a 35% coefficient prevalent among them. Furthermore, many authors highlighted alcohol intake as a risk factor for vascular dysfunction and may adversely affect cochlear microcirculation, thus hindering recovery in patients presenting with SSNHL (15–18). With regard to comorbidities, diabetes mellitus, with a prevalence of 12.50%, and hypertension, with a prevalence of 16.67% in this study, are considered moderate, similar to other investigations (19), where the prevalence of diabetes mellitus was 15% and that of hypertension was 18% among SSNHL patients. Both have been associated with microvascular dysfunction and oxidative stress, which makes the patients have a poor prognosis. Pronounced hearing impairment with SSNHL may have a low genetic component, as only 8.33% of patients noted a family history of hearing loss, which is similar to the results of other conducted studies who found family history in 7% of cases (17–19). Besides, this present study identified occupational noise exposure as the second most common factor that influenced SSNHL risk, with a prevalence of 20.83%, followed by obesity with a prevalence of 15%. This pattern of results supports the claims outlined by other authors, noting that 22% of the participants were exposed to noise and 17% were obese: therefore, noise and obesity ought to be considered modifiable risk factors in the prevention of, and management strategies for, SSNHL (22–26). Some statistical parameters, including confidence intervals and formal model validation metrics, were not fully incorporated, which may limit the robustness of the predictive estimates.

The audiometric patterns and prognosis outcomes for this study are consistent with the previous studies. For audiometric subtypes of HL, high frequency HL (41.67%) was observed more than the low frequency HL (33.33%) and flat or profound HL (25.00%). Of note, the results of the present work align with other studies that recorded high-frequency loss in 40%, low-frequency loss in 32%, and flat loss in 28% (27–29). Audiometric subtype still has an important impact with regard to prognosis, with the flat or profound audiometry indicating a worse prognosis in relation to major cochlear damage. Another prognostic factor that significantly influenced the results was hearing loss severity. The type of hearing loss for this study included mild hearing loss (20.83%), moderate hearing loss (29.17%), severe hearing loss (33.33%), and profound hearing loss (16.67%). In a similar development, previous literature (9–11) recorded the following distribution of hearing loss categories: severe 35% and moderate 28%; mild 22% and profound 15%. In our study, prognosis was significantly associated with the degree of hearing loss; those who experienced severe and profound losses were less likely to regain hearing, as reported by previous authors (12).

This study found that the occurrence and duration of symptoms were mean = 24 h in participants reporting improved outcomes (58.33%). The total number of respondents at 4 weeks was 21; 15 returned to their normal level, 8 had partial recovery, and none of the respondents had no recovery at all. Similar results were presented by other conducted studies (12–14), with the first type in 43%, the second in 35%, and no improvement in 22% of patients. Observed treatment response patterns showed 54.17% significant improvement and more favorable response with corticosteroid and 25.00% improvement with hyperbaric oxygen therapy and 20.83% poor response to treatment. These trends indicate that although corticosteroids continue to be the main form of treatment for the condition, measures such as hyperbaric oxygenation improve the recovery rates for selected patients.

Only blood routine parameters were tested at the baseline and the post-recovery phase in relation to different types of SSNHL – LFHL, HFHL, Flat/Profound Hearing Loss; and the results obtained confirmed the hypothesis on SSNHL as a systemic pathology correlating with the previous research. WBC count was significantly decreased after recovery compared to baseline in all WBC subtypes (WBC_sub): *p* = 0.048; 0.041; 0.045, which indicates resolution of inflammation processes.

MPV also reduced during recovery in all subtypes (*p* = 0.032, 0.028, 0.030), suggesting less activated platelets and inflammation. These outcomes correspond with the findings of other authors who indicated that MPV is a stable sign of systemic swelling reduction after treatment has been made (5–9). RDW also had a significant decrease in all study groups (*p* = 0.026, 0.024, 0.027), showing better erythrocyte homeostasis and decreased oxidative stress as well. This finding is consistent with other studies (4–7) which also explained reduced RDW in patients with significant hearing improvement, stress reduction, and improved oxidative stress. While doing research on the effect of corticosteroid and hyperbaric oxygen therapy on SSNHL patients, mean NLR and PLR were significantly lower after recovery compared to pre-illness state and at admission (*p* = 0.010, 0.008, 0.012 for NLR; *p* = 0.020, 0.015, 0.018 for PLR), implying a low grade of inflammation. The previous research continued to show that the increase in NLR for more than a week was related to poor recovery, confirming the effectiveness of NLR as a prognostic factor on SSNHL (30–32).

Significantly reduced levels of inflammatory markers, such as CRP and ESR, were observed post-recovery across all subtypes (CRP *p* = 0.005, 0.004, 0.006; ESR *p* = 0.015, 0.012, 0.018). These results are associated with a significant reduction of CRP and ESR levels, proving their usefulness as markers of inflammation resolution. Increased levels of CRP and ESR at the beginning of the treatment significantly predicted the poorer recovery of hearing, so these parameters should be considered as important biomarkers. Triglyceride and LDL cholesterol levels were elevated during the disease, while HDL cholesterol declined, and all returned to normal or near normal post recovery. The D-dimer, which reflects thrombotic activity, was significantly reduced after the recovery (*p* = 0.018, 0.015, 0.020), indicating enhanced coical microcirculation. These are in concordance with other studies that also found higher baseline D-dimer in the patients who had worse prognosis of SSNHL (5).

The inflammatory and vascular markers, serum ferritin and FBG, also diminished after recovery to mean values; *p* = 0.012, *p* = 0.010, *p* = 0.014 for ferritin; *p* = 0.022, *p* = 0.020, *p* = 0.025 for FBG. In the same vein, authors noted that hyperfibrinogenemia is associated with a negatively altered cochlear microcirculation, thus a lower rate of recovery (31). We then assessed the metabolic changes after LV using glucose and creatinine for metabolic markers, the results indicated that these two aspects were improved post recovery relative to baseline measures by ANOVA (*p* = 0.020, 0.018, 0.022 for glucose; *p* = 0.030, 0.027, 0.035 for creatinine). Similarly, many authors observed that increased creatinine levels would predict worse auditory prognosis for patients with SSNHL, indicating that metabolic regulation is critical in the SSNHL ear healing process (18–20).

Finally, the albumin level improved in all subtypes compared to baseline (*p* = 0.028; 0.026; 0.033), pointing to an improvement of the general nutritional status and systemic conditions. In the present study, the receiver operating characteristic (ROC) curve analysis showed good evidence of various blood biomarkers in the analysis of prognostication of SSNHL. Among the tested parameters, the NLR offered the highest predictive value with AUC being 0.85 (sensitivity: 82% and specificity: 85%) at a cut-off value of >3.0. In line with other study revealed that NLR was higher in patients with poor recovery rates and inflammation in SSNHL (18). Due to the high accuracy of NLR for predicting the progression of postoperative complications, it can be used in assessing the features of systemic inflammation during routine examinations.

Apart from MMP-9, another significant marker, which included CRP, had an AUC of 0.82%, sensitivity of 78%, and specificity of 80%, with cut-off value of >7. 0 mg/L. This is in line with the study (25) which achieved an AUC of 0.81 for CRP and came to the conclusion that the biomarker was relevant for the prognosis of SSNHL. It seems that these findings have helped establish the role of CRP to indicate the response of SSNHL patients to systemic inflammation, and the potential for using it as an objective marker in prognosis. There were also other significant inflammatory biomarkers that were predicted with great strength, including ESR and PLR. ESR achieved an AUC of 0.79, sensitivity of 75%, and a specificity of 77% using a threshold >15 mm/h; in contrast, PLR had an AUC of 0.80, sensitivity of 76%, and specificity of 79%, with a cut-off of >160. These findings accorded with the study where the ESR and PLR values were reliable markers of disease activity and prognosis to SSNHL patients, with ESR yielding an AUC of 0.77 and PLR of 0.78 (12).

The usage of coagulation markers, such as D-dimer and FBG, was also demonstrated in this study. The overall AUC for D-dimer was 0.74, profiles of sensitivity and specificity at a cut-off >0.35 mg/L being 70 and 73%, respectively, and for FBG, the AUC was 0.75, sensitivity and specificity at a cut-off > 3.5 g/L being 72 and 75%, respectively. These results are similar, after analyzing D-dimer levels in SSNHL patients with poor prognosis, an AUC of 0.73 was presented (12). Similarly, other hematological indices such as neutrophil count had substantial diagnostic accuracy with an AUC of 0.78 and for lymphocyte count with an AUC of 0.72. Peripheral neutrophil count above or equal to 6.5 × 10^9^/L gave a sensitivity of 76% and specificity of 79%, whereas peripheral lymphocyte count below 1.5 × 10^9^/L gave a sensitivity of 68% and specificity of 70%. These findings are consistent with other studies where authors found an AUC of 0.76 in neutrophil count and how this factor may be useful to estimate the inflammatory load in SSNHL (6).

Other metabolic indicators, such as albumin, glucose, and creatinine levels, were included in the current model. Albumin, at the cut-off value of <4.0 g/dL, with AUC 0.73, sensitivity 70%, and specificity 74% corresponds with the result from other authors (20–23), where hypoalbuminemia, defined by an albumin level less than 3.5 g/dL, was significantly associated with worse recovery outcomes (AUC 0.72). Glucose had a 0.71 AUC, 68% sensitivity, and 71% specificity. Creatinine >1.0 mg/dL, which presented an AUC of 0.69, was higher, which was also consistent with the previous author’s study stating that poor SSNHL prognosis is associated with renal dysfunction (2–7). The integration of these findings reveals that inflammation-based prognostic score indicators, such as NLR, CRP, PLR, and ESR, are helpful in predicting SSNHL prognosis, along with coagulation-based prognostic score indicators, D-dimer and FBG, hematological score indicators, neutrophil and lymphocyte counts, and metabolic score indicators, albumin, glucose, and creatinine. These biomarkers, in addition to maintaining a record of systemic inflammation and vascular process, also pointed toward metabolic and immune dysfunction as important determinants of recovery.

Other tested covariate, D-dimer, which is a coagulation biomarker, also showed meaningful prognostic utility (*β* = 0.21, AOR = 1.23, *p* = 0.015). In the same regard, authors pointed out that D-dimer is an independent factor that correlates with delayed hearing resolution, besides being associated with persistent cochlear hypoperfusion (10–14). In statistics of the hematological indices, the study prepared NLR as the most suitable dimension to predict poor prognosis (*β* = 0.41, AOR = 1.51, *p* < 0.001), trailed by PLR (*β* = 0.35, AOR = 1.42, *p* = 0.004). These results are in line other studies that showed that high NLR (>3.0) was unfavorable for the restoration of auditory function because high NLR may reflect persistent systemic inflammation and immune imbalance (18).

Moreover, serum ferritin and FBG levels were other independent predictors of poor prognosis (*β* = 0.29, AOR = 1.34, *p* = 0.007 for ferritin; *β* = 0.25, AOR = 1.28, *p* = 0.011 for FBG). According to the results of the present study, these findings are consistent with the study of other studies, which indicated that serum ferritin levels are high in patients with SSNHL, reflecting increased oxidative stress and systemic inflammation (17–19). Neutrophil count exhibited a positive correlation to recovery outcomes (*β* = 0.31, AOR = 1.36, *p* = 0.003), while the lymphocyte count had a negative correlation (*β* = −0.24, AOR = 0.78, *p* = 0.018). On the same note, in the study conducted by other authors, the balance of lymphocytes would be useful in achieving enhanced SSNHL recovery outcomes (15). Other cell indices were also significant: monocyte count (*β* = 0.22, AOR = 1.25; 95% CI = 1.03–1.52; *p* = 0.022) and eosinophil count (*β* = 0.19, AOR = 1.21; 95% CI = 1.02–1.45; *p* = 0.027).

Different types of hearing loss affected the prognosis differently: Flat/profound hearing loss was the most predictive factor of poor outcomes (*β* = 0.44, AOR = 1.55, *p* < 0.001), followed by high-frequency hearing loss (*β* = 0.26, AOR = 1.30, *p* = 0.008). Treatment given, including corticosteroid (*β* = −0.30, AOR = 0.74, *p* = 0.012) and hyperbaric oxygen administration (*β* = 0.18, AOR = 1.20, *p* = 0.031), was found to predict prognosis. In the same regard, a study on the comparison of effects of combined corticosteroid and hyperbaric oxygen therapy on patient’s auditory outcomes found that there was a positive direction (12).

### Significance of this study

It seems quite appropriate that this study derives pragmatic meaningful insights regarding the applicability of the routinely available blood parameters and clinical and inflammatory markers as predictors of recovery in patients with SSNHL. Hence, in this study, NLR, CRP, ESR, D-dimer, and FBG, are prospects to develop early diagnosis, prognosis, and a proper treatment plan of the disease. Moreover, audiometric patterns, the time of symptom onset, and therapeutic response analysis integration provide clinicians with a fairly detailed approach to decision-making processes. The results are consistent with prior studies documenting that hematological and biochemical markers could be used to predict SSNHL outcomes; the results might be valuable to future research and clinical management.

### Limitations of this study

However, this study has some limitations, which are as follows. The number of patients included in the study for statistical analysis is quite reasonable, but at the same time, it can hardly be considered a representative selection of patients with SSNHL. The observational design restricts researchers’ possibility of drawing causality between the biomarkers being hypothesized and hearing recovery outcomes. Besides, other aspects, including but not limited to heredity, dietary differences, and external stimulants, were given a relatively raw assessment. Other limitations that may have affected the findings include variation in administering treatment plans and patients’ compliance with taking the drugs. Potential confounders, such as dietary factors, medication adherence, and hereditary risk, were not analyzed, which may have influenced biomarker values.

## Conclusion

Consequently, such systemic inflammatory, hematological, and metabolic factors play essential roles in the prognostication and treatment response of SSNHL. For biomarkers, NLR, CRP, ESR, D-dimer, and FBG were identified as significant predictors; also, recovery timing and patterns of audiometric were significant for clinical factors. Early initiation of treatment, along with the time from enrollment to the start of treatment within 24 h, and better management and treatment regimens, including corticosteroids and hyperbaric oxygen, improved prognosis. Incorporation of these predictive parameters in regular clinical measurements could refine the general prognosis estimates and benefit patients’ management. More elaborate studies with greater numbers of patients and longer follow-up must, therefore, be conducted to support the research more powerfully.

## Data Availability

The original contributions presented in the study are included in the article/supplementary material, further inquiries can be directed to the corresponding author.
